# Uterine Hydatids in Virgins

**Published:** 1882-07

**Authors:** 


### UTERINE HYDATIDS IN VIRGINS.

A question of considerable interest was lately discussed before the Dublin Obstetrical Society, to wit: Whether a woman could expel uterine hydatids — in other words, be liable to "molar pregnancy"— without sexual connection. Dr. Moore Madden maintained this to be possible. He thinks the unimpregnated ovule may be arrested in its passage through the uterus, and there undergo a vesicular degeneration or other form of abnormal development. The president, Dr. John A. Byrne, dissented, believing that vesicular moles had not been observed in virgins. He granted, however, that substances not unlike these are occasionally expelled from the virgin uterus. These are not true vesicular chorionic degenerations, as this is always a product of conception.—*New England Med. Monthly.*

